# New Strategies for Preventing Perinatal Group B Streptococcus (GBS) Infections

**DOI:** 10.3390/pathogens15010022

**Published:** 2025-12-24

**Authors:** Dorota Kaminska, Magdalena Ratajczak, Wiktoria Nowicka, Jolanta Dlugaszewska, Marzena Gajecka

**Affiliations:** 1 Chair and Department of Genetics and Pharmaceutical Microbiology, Poznan University of Medical Sciences, Rokietnicka 3, 60-806 Poznan, Poland; dorotakaminska@ump.edu.pl (D.K.); mratajczak@ump.edu.pl (M.R.); wnowicka@ump.edu.pl (W.N.); jdlugasz@ump.edu.pl (J.D.); 2 Institute of Human Genetics, Polish Academy of Sciences, Strzeszynska 32, 60-479 Poznan, Poland

**Keywords:** *Streptococcus agalactiae*, virulence factors, perinatal infections, prevention strategies

## Abstract

Group B Streptococcus (GBS) is a component of the natural human microbiota, colonizing the genitourinary tract and the distal gastrointestinal tract. Due to its production of numerous virulence factors, GBS can cause infections in pregnant women, newborns, and immunocompromised individuals. In newborns, GBS infection may present as severe pneumonia, meningitis, or sepsis. Screening for maternal GBS colonization, combined with intrapartum antibiotic prophylaxis for colonized women, is currently regarded as the most effective strategy for preventing neonatal GBS infections. However, growing concerns regarding antibiotic resistance and the negative impact of antibiotics on the neonatal microbiome have intensified the search for alternative approaches. These include the development of a vaccine and methods to reduce vaginal colonization in pregnant women.

## 1. Introduction

*Streptococcus agalactiae* (Group B Streptococcus, GBS) is part of the human commensal microbiota. They are most commonly isolated from the lower gastrointestinal tract and the urogenital system. Most infections caused by GBS are endogenous, and the mechanism by which the microorganism transitions from a colonizing strain to an invasive pathogen is not yet fully understood [1,2].

According to the CDC, in the United States, 25% to 40% of healthy women are colonized with GBS in their gastrointestinal tract or vagina [3]. GBS colonizes pregnant women and non-pregnant women at a similar rate. In pregnant women, there is a significant risk of transmission of the microorganism to the baby during childbirth. Statistics show that about 50% of newborns born to GBS-colonized women also become colonized with the bacteria, but only 1–2% of these newborns develop an infection. Infections in newborns can manifest as pneumonia, meningitis, or sepsis [4].

In pregnant and postpartum women, *S. agalactiae* can cause asymptomatic bacteriuria during pregnancy, preterm labor, intrapartum fever, amniotic fluid infections, and sepsis. However, GBS is not only an etiological agent of perinatal infections. In non-pregnant adults, particularly the elderly, individuals with weakened immunity, and those with comorbid conditions (such as diabetes, heart disease, neurological disorders, kidney disease, and obesity), GBS can cause skin and soft tissue infections, pneumonia, endocarditis, arthritis, osteomyelitis, peritonitis, and sepsis. It is also an etiological agent of urinary tract infections [1,5].

Perinatal infection prevention includes screening of pregnant women and antibiotic prophylaxis during labor. Due to the growing antibiotic resistance and the adverse effects of perinatal antibiotic therapy on the neonatal microbiome, new methods of preventing GBS infections are being sought. Efforts are focused on the development of a vaccine and on finding methods to reduce vaginal colonization in pregnant women.

We discuss issues related to new methods of GBS prevention, including the importance of epidemiological monitoring of GBS serotypes and their surface proteins in a given geographic region, as this plays a significant role in the vaccine design process and the implementation of future vaccination strategies. We also present the latest findings on the interactions between the vaginal microbiota and *S. agalactiae*, which may contribute to the development of new strategies for limiting colonization in women through the use of probiotic bacteria. Furthermore, reports suggesting the potential use of photodynamic therapy to reduce GBS colonization are discussed.

## 2. Diseases Caused by GBS in Neonates and Associated Risk Factors

The incidence and clinical manifestations of GBS infections vary across patient populations and geographical regions. Maternal colonization of the genitourinary and lower gastrointestinal tracts during the perinatal period is the primary source of neonatal GBS infections, which can occur in utero, during delivery, or postnatally. Based on the timing of symptom onset, GBS infections in neonates are classified into early-onset disease (EOD), late-onset disease (LOD), and late-late-onset disease (LLOD) [6,7].

EOD is characterized by the presence of bacteria in the blood, cerebrospinal fluid, or other usually sterile sites in the newborn by the sixth day of life. EOD typically manifests as acute pneumonia, respiratory failure, sepsis, and meningitis. Neonatal exposure to GBS may occur several hours to days prior to birth or during passage through the birth canal. Transmission pathways include ascending infection from the uterine cavity, vertical transmission from mother to fetus, and peripartum exposure during delivery. The main cause of EOD is GBS colonization of the mother’s urogenital and lower gastrointestinal tracts. Other risk factors include a gestational age of less than 37 weeks, young maternal age, very low birth weight, maternal African American race, premature rupture of membranes, and intra-amniotic infection [6,8,9].

Aspiration and swallowing of infected secretions from the genital tract during delivery cause the bacteria to adhere to the mucous membranes of the newborn’s respiratory and gastrointestinal tracts, leading to colonization. If the infant has a poor antibody response (transferred from the mother) or immune system disorders, or if the bacteria have virulence factors, bacterial invasion into the bloodstream may occur. Moreover, the lower the gestational age, the weaker the defense via opsonins mediated by neutrophils in the newborn. It has been shown that preterm infants have lower levels of protective maternal antibodies compared to full-term infants. Premature rupture of membranes is also a frequent cause of EOD infections, as it facilitates ascending colonization, allowing bacteria to enter the amniotic fluid. A significant factor suggesting intra-amniotic infection is intrapartum fever, which reflects the maternal response to ongoing bacterial infection [6,8,9].

LOD is characterized by the presence of bacteria in the blood, cerebrospinal fluid, or other usually sterile sites between the seventh and 89th day of life, while LLOD has an onset between 90 days and 6 months of age. Both clinical entities manifest as sepsis and meningitis [10,11]. Newborns can be colonized either through maternal transmission or through non-maternal sources during the perinatal and postpartum periods. A positive GBS screening test result during pregnancy is associated with an increased risk of infection. Other risk factors include preterm birth, multiple births, ethnic background (Black women), and young maternal age. Intrapartum antibiotic prophylaxis (IAP) aims to reduce colonization during birth, but in the case of LOD, a significant number of newborns acquire the pathogen after birth. It has been proven that IAP does not reduce the incidence of LOD in newborns [8,9].

There have been reported cases of GBS transmission through breast milk [7,12]. A mother infected with GBS, whether or not she has mastitis, is considered a potential carrier of the pathogen. Another theory suggests that the newborn acquires GBS while passing through the birth canal, which colonizes the baby’s oral cavity and throat. During breastfeeding, the pathogen is transmitted and infects the mother’s mammary glands. This leads to bacterial overgrowth in the breast, which reinfects the child. In addition to the colonized mother, the source of the pathogen can also be caregivers or healthcare workers who transmit the microorganism. Transmission can also occur due to factors such as hospital beds being too close together, insufficient surface and equipment disinfection, and a shortage of nurses relative to the number of patients. Inadequate treatment of GBS infection episodes, immune deficiency, and increased bacterial virulence can result in recurrent infections [13].

Group B streptococci colonizing the genital tract of pregnant women can contribute to postpartum endometritis, wound infections, urinary tract infections, and sepsis. Secondary complications, such as bacteremia, meningitis, osteomyelitis, or endocarditis in pregnant women, are rare.

## 3. Epidemiology of GBS: Serotype Distribution and MLST Sequence Types

The classical tool used for epidemiological purposes is the capsular serotyping of GBS. Capsular polysaccharide (CPS) is one of the most important virulence factors, which is rich in sialic acid [2]. CPS affects the durability and survival of GBS in the host’s body. It mimics host carbohydrate epitopes, allowing GBS to evade immune system actions. CPS also interferes with complement-mediated defense mechanisms and neutrophil phagocytosis and facilitates intracellular survival of the bacteria within dendritic cells [14]. Based on differences in the structure of the CPS, 10 serotypes of *S. agalactiae* have been identified, designated as Ia, Ib, II, III, IV, V, VI, VII, VIII, and IX. Serotype IX was the last to be described, in 2007 [15]. The prevalence of individual capsular serotypes varies by geographic region (Table 1).

It has been found in a meta-analysis that 97% of invasive GBS strains isolated from newborns belong to five serotypes (Ia, Ib, II, III, and V) [22]. Among the mentioned serotypes, serotype III appears to be the most significant, as it is responsible for the majority of invasive infections in newborns. However, it is not the only one relevant to neonatology and pediatrics. Weisner et al. assessed the prevalence of particular serotypes in strains isolated from invasive infections in newborns in England and Wales, identifying serotype III as the most common (48%), followed by Ia (27%) and V (10%) [23]. The authors observed a difference in the distribution of serotypes in isolates from patients with EOD, where serotype III accounted for 38%, Ia for 32%, and V for 13%, compared to LOD, where serotype III accounted for 67% [23].

Salloum et al. determined the serotype distribution in 142 *S. agalactiae* strains isolated from invasive infections in adults (n = 75) and newborns (n = 67) in France (24). The strains responsible for infections in newborns were primarily of serotype III (79%) and Ia (12%). In contrast, among the strains isolated from adults, serotype V was predominant (31%), followed by Ia (23%), III (19%), and Ib (19%) [24].

The high heterogeneity of serotypes and their varied distribution pose a significant challenge in research aimed at developing an effective vaccine. Therefore, epidemiological studies are crucial, as they will provide a detailed understanding of the distribution of serotypes and enable further work on effective GBS infection prevention strategies.

With the development of genetics and molecular research, new tools, such as Multilocus Sequence Typing (MLST) of seven genes, have been introduced for classifying GBS. Strains with different serotypes may have the same MLST sequence type (ST). The capsular serotype is not limited to a specific ST. Several STs are grouped into clonal complexes (CC) when they share six or seven matching alleles. Using MLST, most human GBS isolates can be grouped into six clonal complexes: CC1, CC19, CC17, CC10, CC23, and CC26 (14). It has been observed that serotype III strains with ST-17 sequences are highly hypervirulent and are responsible for the high incidence of invasive diseases in newborns [2,25,26]. In Slovenia, it was observed that isolates of serotype III were predominantly associated with invasive disease (present in 74% of cases with the invasive isolates) and belonged to ST-17 (52%) [25]. Italian researchers obtained similar results, with serotype III accounting for 57.7% of early-onset disease (EOD) cases and most late-onset disease (LOD) cases (93.6%). Majority of these strains, isolated from both EOD and LOD cases, belonged to ST-17 (26). The presence of the ST-17 clone has also been confirmed in Polish population [27,28].

## 4. Virulence Factors That Promote GBS Vaginal Colonization and Infection

*S. agalactiae* persists as an asymptomatic colonizer, primarily in the female rectovaginal tract. Several strategies used by GBS to colonize its host have been recognized, including the bacterial binding to host epithelial cells and weakening the host’s immune defenses. Some GBS strains exhibit increased pathogenic potential due to the acquisition of specific virulence factors, which enhance the bacteria’s ability to spread, damage host tissues, and evade immune responses more effectively.

In addition to the capsular polysaccharide, the most important factors determining the virulence of GBS strains are regulatory proteins, surface proteins, and the toxins they produce. All these components form a complex system based on interrelationships, designed to enable the pathogen to function within the host organism. For the microorganism to persist in the body, its adhesion to host cells and extracellular matrix proteins is essential. Therefore, GBS has developed a variety of proteins known as adhesins, which are present on the surface of the bacterial cell. These adhesins facilitate the binding of the microorganism to components of the human epithelium, thus enabling colonization. The most important adhesins include fibrinogen binding proteins (FbsA, FbsB, FbsC, BsaB, Srr1 and Srr2), laminin binding protein (Lmb), plasminogen binding surface protein (PbsP), C5a peptidase (ScpB), hypervirulent GBS adhesin (HvgA), and GBS immunogenic bacterial adhesin (BibA) [28,29,30,31,32].

Strains with ST-17 sequences carry various virulence factors, including a surface-anchored protein known as hypervirulent GBS adhesin (HvgA). The presence of this adhesin promotes adhesion to intestinal epithelial cells, choroid plexus epithelial cells, and microvascular endothelial cells that constitute the blood–brain barrier [33]. The *hvgA* gene, which encodes this adhesin, is effectively utilized for identifying ST-17 strains. Recent epidemiological data suggest the spread of a multidrug-resistant ST-17 GBS subclone, which is characterized by the loss of PI-1 [CC (clonal complex) 17/PI-2b], and is simultaneously resistant to macrolides, lincosamides, tetracycline, and other antibiotics [26,28,34,35].

In addition, surface structures, pili (encoded by genomic islands PI-1, PI-2a, and PI-2b), are considered as essential adhesins in promoting GBS colonization, persistence, biofilm production, and central nervous system invasion. Each variant has a different function in the pathomechanism of infections. PI-1 protects the microorganism from macrophages and phagocytes by masking it in the host, allowing the pathogen to operate freely while immune cells mistakenly do not recognize it as a threat. PI-2a plays a crucial role in bacterial adhesion to the epithelium and biofilm formation, while pilus PI-2b is responsible for the invasion of host cells. Mutations within the *pi2b* gene result in significantly reduced ability of the pathogen to colonize epithelial cells, thus reducing the incidence of GBS infections. Additionally, the presence of Pl-2b on the cell surface plays an essential role in CNS invasion, enabling streptococci to cross the blood–brain barrier [36,37,38].

GBS produces various toxins like hemolysins, hyaluronidases, superoxide dismutase, and the CAMP factor, which help the pathogen invade, survive, and spread within the host [2,39,40,41,42].

Major GBS virulence factors, with their specific targets and functions, are summarized in Table 2.

*S. agalactiae*, like many other pathogens, has the ability to form biofilm. This structure allows GBS to be an excellent colonizer, adapting well to various environments. Besides reducing susceptibility to pharmacological agents, biofilm structure enables the pathogen to evade the immune response by masking it within the host organism [48].

In biofilm formation, the pilus PI-2a plays an important role. Studies on strains with disrupted pilus structure or defective enzymes crucial for its proper function showed significantly reduced adhesion to host epithelial cells and thus a decreased ability to form biofilm. This was also confirmed using confocal laser microscopy, which allowed for comparison between strains with a fully functional pilus and strains with structural defects. The results clearly demonstrated a greater capacity for forming multilayered bacterial aggregates, resembling mature biofilm, among GBS strains with properly formed PI-2a protein. Strains with defective PI-2a protein showed a much lower capacity for biofilm production [49,50].

High variability between strains and diversity in pilus expression on the cell surface lead to unequal biofilm production, even within the same serotype. Research has shown that isolates most frequently forming biofilm belong to the hypervirulent serotype III. The exact reasons for this phenomenon remain unclear, as do the protein structures that enable this strain to colonize epithelial cells more effectively. Uncovering these mechanisms would facilitate efforts to control infections caused by the ST-17 strain. Differentiating GBS strains by their biofilm formation capacity, depending on their origin, has shown a greater tendency for biofilm formation among isolates obtained from newborns [5,51,52].

From the perspective of infection treatment, biofilm formation is an unfavorable phenomenon, so it is crucial to develop strategies to block its formation. Research indicates that biofilm production can potentially be inhibited by immunoglobulins targeting specific components of the PI-2a protein. This offers hope for new therapeutic approaches and infection control strategies for GBS biofilm.

Summarizing, the virulence of GBS results from the complex interaction of several factors. The polysaccharide capsule, adhesive proteins, and tissue-degrading enzymes, as well as the ability to form biofilms and evade phagocytosis, enable this bacterium to colonize the host and cause infections. Understanding these mechanisms is crucial for developing new treatment and prevention strategies for infections caused by GBS.

## 5. Perinatal GBS Prophylaxis

Infections caused by GBS are a significant concern in perinatal care. GBS can lead to severe neonatal infections such as sepsis, pneumonia, or meningitis, especially in the first week of life. To address this risk, some strategies have been developed to reduce the likelihood of GBS transmission from mother to child. The CDC, in collaboration with organizations such as the American College of Obstetricians and Gynecologists (ACOG), developed a set of guidelines aimed at reducing perinatal GBS infections [3,4]. It is known that the primary risk factor for early-onset neonatal infection is maternal colonization of the lower gastrointestinal and genitourinary tracts, which can lead to transmission during labor or following membrane rupture just before delivery.

Perinatal prophylaxis includes screening pregnant women for GBS and intrapartum antibiotic prophylaxis (IAP). Screening is recommended between 35 and 37 weeks of pregnancy. Vaginal and rectal swabs are taken and cultured for GBS. Women testing positive (GBS-positive) are candidates for IAP during labor [3,4].

Intrapartum antibiotic prophylaxis is administered during labor to women identified as GBS-positive or meeting specific risk criteria (e.g., previous delivery of a baby with GBS infection, GBS bacteriuria during pregnancy). Penicillin G is the first-line drug, with ampicillin as an alternative. For women allergic to penicillin, options include cefazolin, clindamycin, or vancomycin, depending on GBS susceptibility testing. In accordance with recommendations, antibiotics are given intravenously at least 4 h before delivery. Prophylaxis is particularly recommended for preterm births (<37 weeks), prolonged rupture of membranes (>18 h), or intrapartum fever. In some countries, a risk-based approach is used instead of universal screening (e.g., preterm labor, intrapartum fever). However, this approach is less effective in reducing EOD cases [3,4].

The introduction of screening and IAP has reduced the risk of EOD by over 80%. However, prophylaxis does not prevent late-onset disease (LOD), which occurs after the first week of life and involves different transmission mechanisms [4].

## 6. Antibiotic Resistances of GBS

The drug of choice for treating *S. agalactiae* infections is penicillin. However, analysis over recent years shows a decrease in sensitivity to penicillins and cephalosporins among GBS strains. For women who report a high risk of anaphylactic reactions to penicillin, clindamycin, a lincosamide antibiotic, is used for peripartum prophylaxis. Therefore, the growing resistance of GBS to macrolides, lincosamides, and streptogramin B poses a significant problem [53].

Resistance of GBS to macrolide, lincosamide, and streptogramin B (MLS_B_) antibiotics can result from two primary mechanisms. The first mechanism involves modification of the drug’s target site, which is mediated by ribosomal methylase genes (*erm*). Over 20 classes of *erm* genes have been described, with *ermB* and *ermTR* being the most common in *Streptococcus* spp. These genes can confer resistance via both inducible and constitutive MLS_B_ mechanisms. Inducible MLS_B_ resistance occurs when there is phenotypic resistance to erythromycin, but the susceptibility to lincosamides such as clindamycin and lincomycin is retained. Constitutive MLS_B_ resistance is present when there is simultaneous resistance to erythromycin and clindamycin. In cases of both inducible and constitutive MLS_B_ resistance, the use of macrolides, clindamycin, and streptogramin B antibiotics is not recommended, as it poses a high risk of therapeutic failure.

The second mechanism is active drug efflux through a transmembrane pump, mediated by *mef* genes, typically *mefA* and *mefE* in *Streptococcus* spp. These genes confer resistance only to 14- and 15-member macrolides, while bacteria with these genes remain susceptible to 16-member macrolides, streptogramins, and lincosamides. This resistance profile corresponds to the M phenotype. Additionally, an L phenotype has been identified, which is characterized by resistance to lincosamides like clindamycin but susceptibility to macrolides such as erythromycin. The L phenotype is rare in *S. agalactiae* and is caused by modifications involving the *lnu* gene family. These genes encode nucleotide transferase enzymes that adenylate lincosamides such as clindamycin and lincomycin. Moreover, resistance to tetracycline is encoded by ribosome-protection genes (*tetM* and *tetO*) as well as efflux pump genes (*tetK* and *tetL*) [54,55].

Studies conducted in various parts of the world have pointed to the emergence of multidrug-resistant *S. agalactiae* strains belonging to the ST-17 clone [26,28,56]. This clone contains conjugative and integrative elements, which have contributed to its resistance to tetracycline, lincosamides, macrolides, and high resistance to aminoglycosides. This resistance may be due to the loss of pilus islands, particularly Pl-1, in the bacteria. In the Italian population, it was noted that the loss of Pl-1 was associated with the *ermB* and *tetO* genes, suggesting that these bacteria acquired multidrug resistance [26]. Similarly, in Saudi Arabia, analysis revealed that serotype III strains with ST-17 were strongly associated with Pl-2b, which could increase their infective potential and reduce host immune detection. In other serotype III strains lacking ST-17, different variants of Pl-1 and Pl-2a were present [56].

Research data from various geographical regions has shown a substantial rise in resistance to macrolides and lincosamides. In Italy, resistance at 31.2% for erythromycin and 40.1% for clindamycin has been reported [57]. This increasing trend is also observed in countries outside Europe, such as Iran, Garakuwa in South Africa, and Saudi Arabia [56]

Data from European countries such as Italy and Poland indicate the dominance of the constitutive MLS_B_ resistance phenotype in *S. agalactiae*, with frequencies of 77.7% and 76.4%, respectively [26]. Similar results were reported based on studies conducted in other parts of the world. In Saudi Arabia, the prevalence of the constitutive MLSB phenotype was 55.42%; in South Africa, it was 69%; and in Iran, it was 62% [56]. The phenotypic distribution in various studies was found to be diverse, as detailed below. In the Italian population, the phenotypes were distributed as follows: M phenotype (19.7%), induced MLS_B_ phenotype (2.6%), and no L phenotype detected (26). Similarly, in the Polish population, the distribution was M phenotype (14.6%), induced MLS_B_ phenotype (6.74%), and L phenotype (2.25%) (28). In Saudi Arabia, the phenotypic distribution differed: induced MLS_B_ phenotype (55.42%), L phenotype (9.64%), M phenotype (1.2%) [56].

Resistance to tetracycline is frequently detected in *S. agalactiae* strains. A report from Slovenia indicated tetracycline resistance at 87.2% [25]. Researchers in other parts of the world have also reported similarly high resistance rates [56,58].

## 7. New Prevention Strategies, GBS Vaccine and Probiotic Strategies Development

IAP has led to a decrease in the incidence of EOD in countries where this strategy has been implemented. However, there are places where, for various reasons, such as economic factors, it is not widely used. Additionally, IAP is not effective enough, as it has little effect on reducing the incidence of LOD, postpartum diseases in women, and infections in preterm infants. This prophylaxis is also not applicable to adults with comorbidities or weakened immune systems, who are more susceptible to GBS infections. The use of this strategy is also associated with the development of antibiotic resistance, changes in the microbiome of both mother and child, and the long-term consequences that follow [59].

Antibiotics taken by mothers reach the fetus’s bloodstream through the umbilical cord and may have an effect for at least ten hours after administration, likely influencing early microbiota colonization, but may also contribute to changes in the maternal vaginal and gut microbiomes, which may consequently influence vertical microbial transmission and infant immunity after birth [60]. Despite extensive microbiome research in recent years, the impact of antibiotics administered during pregnancy on neonatal gut colonization and health remains largely unknown [61]. The use of prenatal antibiotic prophylaxis to prevent perinatal GBS infection affects the neonatal gut microbiota by reducing the number of *Bacteroidetes* and *Actinobacteria* and increasing the number of Proteobacteria, thus altering the undisturbed infant colonization pattern. The development of a healthy gut microbiota during infancy is essential because it plays an important role in, among other things, the maturation of our immune system [61,62]. IAP could alter the neonatal gut microbiota by reducing both the abundance and diversity of bifidobacteria, including *Bifidobacterium breve*, *B. bifidum*, and *B. dentium* [61,63]. It is likely that a combination of factors, such as a cesarean section and IAP or formula feeding and IAP, affects the microbiota and prolongs the duration of altered microbial colonization patterns in neonates [62,64]. Arboleya et al. observed low impact of IAP on gut microbiota in neonates in the first days after birth, but some disturbances became evident by one month of age, with most alterations resolving by the age of three months [61]. Nevertheless, the long-term health consequences of IAP-induced microbiota changes remain to be elucidated.

Therefore, there is a need to develop alternative strategies to avoid GBS colonization during pregnancy. Introducing a GBS vaccine could effectively increase protection against infection for individuals who do not receive IAP. The use of probiotics, which have proven antagonistic effects against GBS, is also promising.

### 7.1. Vaccination of Pregnant Women Against GBS

The development of an effective vaccine against *S. agalactiae* administered to pregnant women has been identified as a priority in 2015 by the WHO Product Development for Vaccines Advisory Committee (PDVAC) to protect against the neonatal group B streptococcal infections, especially the late form of this infection, in which antibiotic prophylaxis in pregnant women is ineffective [65,66,67]. It is estimated that after vaccine introduction, it will also be possible to prevent approximately 23,000 stillbirths, 185,000 preterm births, 127,000 early-onset and 87,300 late-onset infant cases of *S. agalactiae* infection [67,68]. Additionally, a vaccine administered to the mother during pregnancy could reduce the need for intrapartum antibiotic prophylaxis, which may contribute to the development of antimicrobial resistance and disrupt the development of the infant’s microbiome. A vaccine would be beneficial in preventing group B streptococcal infections for all women worldwide, but especially for those living in resource-limited societies where microbiological testing and the previously mentioned intrapartum antibiotic prophylaxis are unavailable [69].

Assuming that a vaccine against *S. agalactiae* proves to be an effective form of prevention, intensive research has been conducted since the 1980s to develop a suitable preparation; however, currently, there are no registered or available vaccines against GBS. Several preparations are in the clinical trial phase [68,70,71]. Baker and Kasper in 1976 suggested the transplacental transfer of maternal antibodies against GBS and their role in preventing infection of the newborn [70,72]. Placental transport of immunoglobulin G (IgG) begins around 17 weeks of gestation and increases as pregnancy progresses, with fetal IgG levels being higher than maternal serum levels observed at 40 weeks of gestation. Factors such as placental abnormalities, total maternal IgG concentration, vaccine type, and the interval between vaccination and birth may influence the transplacental transport of vaccine-specific antibodies [73]. Vaccinating mothers is already an effective way to prevent influenza, tetanus, diphtheria, polio and pertussis in infants. Vaccine administration to women during the second or third trimester of pregnancy protects the fetus and, subsequently, the newborn through transplacental transfer of maternal antibodies [73].

To date, two types of vaccines have been developed and tested, namely polysaccharide conjugate vaccines and recombinant protein vaccines, which contain immunogenic domains of bacterial surface proteins [74]. The purified capsular polysaccharide of *S. agalactiae* was unable to induce a sufficient immune response in adults, so it was conjugated with chemically detoxified diphtheria and tetanus toxin (TT) or genetically detoxified diphtheria toxin (CRM197), thereby intensively stimulating immune cells [66]. However, increased vigilance is required, as some GBS conjugate vaccines may interact with other conjugate vaccines such as pneumococcal, meningococcal and influenza B vaccines [75].

The development of multivalent pneumococcal conjugate vaccines (PCVs) directed against capsular polysaccharides has intensified work on multivalent polysaccharide vaccines against the most common capsular serotypes of *S. agalactiae* (Ia, Ib, II, III, IV, and V) [66,74,76]. Strains with capsular polysaccharide type III account for 61.5% of invasive GBS disease cases worldwide [77]. The first clinical trials were conducted using monovalent vaccines (Ia, Ib, II, III and V); however, single serotypes did not stimulate cross-immunity against other serotypes. This situation initiated work on a multivalent vaccine [65]. The trivalent vaccine (Ia, Ib and III) conjugated to CRM197 was evaluated in a group of pregnant and non-pregnant women and was characterized by a high level of safety, satisfactory induction of the immune response and transplacental transfer of IgG antibodies [66]. This paved the way for a pentavalent vaccine (Ia, Ib, II, III and V), also conjugated to CRM197, which began clinical trials in 2017 [66]. It was expected to be effective in the United States, Europe and Australia because it covered the five main serotypes circulating in these areas [70]. Due to the increasing share of serotype IV in infections caused by GBS, Pfizer designed a maternal 6-valent CPS–cross-reactive material 197 (CRM197) glycoconjugate vaccine (GBS6), whose phase three clinical trials were to begin in 2023, and which was to cover as much as 98% of isolates causing invasive neonatal disease [65,66]. Ultimately, phase two of these studies was completed in 2024. One of the last results was submitted by Pfizer to ClinicalTrials.gov in June 2025, and so far there is no information about the start of the next phase of the study, and the mentioned results must be subjected to Quality Control [78]. Additionally, phase 2b studies were conducted in healthy, non-pregnant adult women to assess whether concomitant administration of vaccines against the tetanus, diphtheria, and acellular pertussis (Tdap) and GBS6 targeting serotypes Ia, Ib, II, III, IV, and V causes immunological interference [76]. These results confirm that GBS6 co-administered with Tdap vaccine was well tolerated. The immune response induced by Tdap vaccine co-administered with GBS6 was similar to that induced by Tdap vaccine alone for tetanus and diphtheria antigens. However, the immune response to pertussis antigens was lower with GBS6 co-administration, which is not fully understood, as a correlate of protection for pertussis vaccines has not yet been established. The immune response induced by GBS6 for serotypes Ia, III, and V following co-administration of Tdap vaccine was lower, and for GBS6 serotypes Ib, II, and IV, it was higher, compared to the immune response induced by GBS6 alone. These results may contribute to further clinical trials of GBS6, this time in pregnant women, which may lead to changes in vaccination programs for pregnant women [76]. Although clinical trials have demonstrated the immunogenicity of GBS vaccine candidates, no Phase III clinical trials on the efficacy of any of the vaccines described above have been conducted to date [67]. Another candidate is a multivalent vaccine developed by PATH and Inventprise, which is in the first phase of clinical trials [68].

Protein vaccines may be a better alternative to polysaccharide vaccines because bacterial proteins likely represent a more universal target, ideal for vaccine development [70,74]. Several protein vaccines are in preclinical testing, but the vaccine developed by Minervax is currently in the second phase of clinical trials to assess its safety and immunogenicity in pregnant women. This vaccine targets the N-terminal domain of the alpha-type surface protein family (GBS-NN) [68]. The results of the first-phase trial were very optimistic. Vaccinating healthy women with one or two doses increased antibody levels by more than 30 times [65]. In 2022, GBS6 and GBS-NN vaccine candidates received PRIME status from the European Medicines Agency (EMA), which is related to the great demand for these preparations in medicine [68].

The possibility of developing a common vaccine against *S. agalactiae* and *S. pneumoniae* was previously suggested because the administration of a preparation based on unconjugated GBS type III polysaccharide (IIIPS) or conjugated GBS type III polysaccharide covalently bound to tetanus toxoid (III-TT) resulted in opsonization of both GBS III and *S. pneumoniae* type 14 (Pn14). Additionally, attempts were made to create a multi-component, cross-species vaccine using pili, which are protective antigens in *S. pneumoniae* and GBS. However, it should be remembered that the implementation of pan-pathogenic vaccines, protecting against many mucosal pathogens, must be combined with the assessment of their impact on the natural microbiota [75].

GBS serine-rich repeat glycoprotein is treated as a vaccine candidate due to its conservation; it was detected in 86.7% of clinical isolates. The effect of vaccinating mice with a preparation based on Srr1 containing the latch domain was their protection against *S. agalactiae* meningitis [79]. Another GBS virulence factor considered in the context of developing a protein vaccine is C5a peptidase. C5a peptidase encapsulated within microspheres consisting of a copolymer of lactic acid and glycolic acid induced an immune response in mice, thus protecting them against infection [65]. Other studies have shown the possibility of using four potential biomarkers to develop a vaccine against GBS, which are thioredoxin, CsbD-like protein, RpL7/L12 and exoDNase [75].

Despite many years of work on the best vaccine against *S. agalactiae*, some problems remain unsolved. A matter of debate is the number of doses that should be administered to obtain the desired protection for the child. Few publications have dealt with this issue. One study did not show an increase in antibody levels after the second dose was administered to non-pregnant women one month after the first dose of the trivalent CRM conjugate vaccine, while the results of another study published in 2019 suggested that the time that had passed from the first dose was important in this case. Administration of the second dose of the trivalent (Ia, Ib and III) CRM conjugate vaccine to non-pregnant women 4–6 years after vaccination with the first dose increased antibody levels ≥ 200-fold. Women may need to take an additional dose in future pregnancies [65]. In high-income countries, where IAP is the established standard of care, *S. agalactiae* vaccine could be used as a complementary public health tool, whereas in the low- and middle-income countries, it could provide substantial benefits in reducing the invasive GBS disease [66]. In South Africa, vaccination against *S. agalactiae* would prevent up to 54% of infant infections, saving $676 to $2390/disability-adjusted life-year at a cost of $10–$30 per vaccine. It has been estimated that vaccinating women in West Africa with the hexavalent vaccine could prevent 55% of GBS disease cases and more than 700 years of life associated with disability, compared to no *S. agalactiae* prevention. Achieving 70 percent effectiveness through vaccine use would cost a total of $12. The prevalence of *S. agalactiae* proved to be the most important parameter in the cost-effectiveness relationship estimations [66]. According to Trotter et al., vaccinating approximately 99 million pregnant women in several countries could cost $1.7 billion but would save approximately $300 million in acute infection treatment costs and $85 million in long-term healthcare costs for pregnant women and newborns [66,80]. Kim et al. estimated that vaccinating mothers alone would cost less than screening combined with prophylactic antibiotics, but to prevent the disease, the vaccine’s effectiveness must be almost 90% [71]. Another difficulty in the development of GBS vaccines is the limited sensitivity of tests that quantify Ig isotypes [75]. The ELISA technique cannot be successfully used for this purpose because it results in either non-specific binding of the immobilized CPS to the solid phase or serotype-independent binding of lower avidity antibodies. Additionally, the optimal concentration of antibodies needed to protect the infant throughout the risk period is unknown [65]. To date, regulatory authorities have not agreed on any licensing pathways for vaccines against *S. agalactiae*. In clinical trials, potential interference with other vaccines administered during pregnancy and with the routine vaccination schedule should also be assessed, particularly for the polysaccharide-protein conjugate vaccines administered in the routine schedule [66].

There are legitimate concerns about polysaccharide vaccines that formulations targeting only a limited number of polysaccharide serotypes may lead to their rapid change, as was the case with the pneumococcal vaccine mentioned above [74]. Intra- and inter-species horizontal gene transfer (HGT) can lead to the development and expansion of GBS strains with novel capsule structures, potentially leading to vaccine ineffectiveness. It is hypothesized that the HGT could generate a greater diversity of capsular serotypes in the future, significantly increasing serotypes’ diversity. Surveillance for such events is warranted [81]. Developing a single, universal vaccine is also a challenge, as preparations based on serotypes common in one area may not cover common serotypes in another area due to geographic variations observed even at the regional level. For this reason, vaccine development must be accompanied by strict surveillance of *S. agalactiae* isolates worldwide [70]. Furthermore, the increasing involvement of non-encapsulated strains in the development of infections forces the search for another target for developing an effective vaccine [75]. Attempts are being made to identify proteins common to all GBS strains so that the created vaccine protects against all *S. agalactiae* serotypes. For this purpose, the JUNO project was established; it aims to sequence the whole genome of GBS isolates from a wide geographical and temporal range and find the best target for a vaccine [82].

### 7.2. Probiotics in the Reduction of GBS Colonization

The vaginal microbiome is a dynamic and microbially rich ecosystem that plays a crucial role in women’s urogenital health. In healthy women of reproductive age, in the genital tract, *Lactobacillus* spp. and other bacterial genera, including *Corynebacterium*, *Gardnerella*, *Streptococcus*, *Mycoplasma*, *Escherichia*, *Staphylococcus*, *Mobiluncus*, *Prevotella*, *Atopobium,* and *Megasphaera,* are being recognized [83,84,85,86].

**Figure 1:**
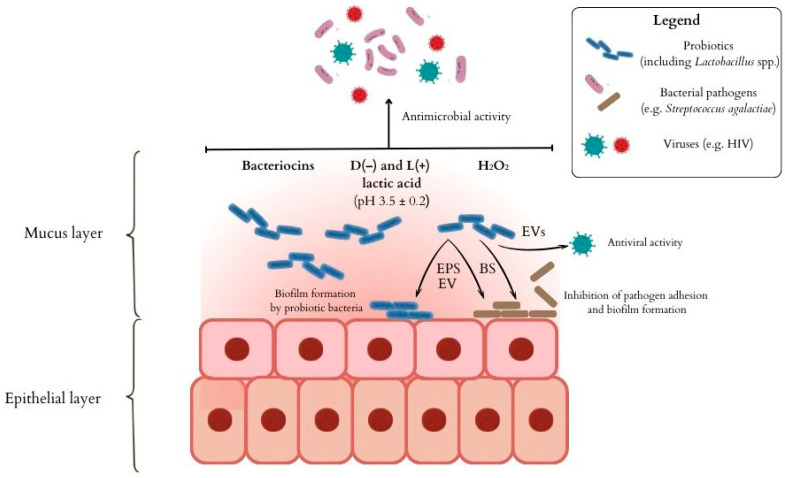
The influence of probiotic bacteria, including *Lactobacillus* spp., on maintaining the balance of the vaginal microbiota. Probiotic bacteria in anaerobic metabolism produce L- and D-lactic acid, which provides an acidic vaginal environment, inhospitable to bacterial, fungal and viral pathogens [91,95]. Bacteriocins and hydrogen peroxide (H_2_O_2_) are substances with antimicrobial activity [95]. Other metabolites of lactic acid bacteria, such as biosurfactants (BS), exopolysaccharides (EPS), or extracellular vesicles (EVs), impede the adhesion of pathogens to human cells and the formation of biofilm. EPS and EVs also stimulate the formation of biofilm by probiotic bacteria, and EVs additionally impair viral infection [91].

*Lactobacillus* species have the potential to maintain vaginal homeostasis through several mechanisms: the production of lactic acid and other antimicrobial compounds, regulation of local immune responses in the cervical and vaginal mucosa, and competition with other bacteria for ecological niches, which hinders the expansion of other bacteria (Figure 1) [87,88,89,90]. In addition, *Lactobacillus* fights against GBS biofilms through multiple strategies, including lowering pH, producing biosurfactants or exopolysaccharides, competing for space and nutrients, blocking adhesion sites, interfering with *quorum sensing*, and altering GBS gene expression (like inhibiting biofilm-related genes), making surfaces unfavorable for GBS colonization and biofilm maturation [91,92,93,94].

Both in vitro and in vivo studies have demonstrated that *Lactobacillus* species inhibit the adhesion and growth of various urogenital pathogens, including group B *Streptococcus*, *Staphylococcus aureus*, *Gardnerella vaginalis*, *Neisseria gonorrhoeae*, and *Escherichia coli* [96,97,98]. Additionally, some *Lactobacillus* species form a thick protective biofilm, which enhances the stability and resilience of the vaginal microbial community [99,100].

Marziali et al. evaluated the activity of 14 *Lactobacillus* strains (belonging to species *L. crispatus*, *L. gasseri*, and *L. vaginalis*), isolated from the vagina, against GBS isolates [96]. They assessed the ability of both *Lactobacillus* cells and their culture supernatants to reduce GBS viability. It was observed that the acidic environment produced by *Lactobacillus* bacteria is crucial in inhibiting GBS growth. Additionally, certain *Lactobacillus* strains demonstrated the ability to inhibit the adhesion of GBS to vaginal epithelial cells under in vitro conditions [97,98]. Ephraim et al. investigated the antagonistic effects of *Lactobacillus rhamnosus* HN001 and the probiotic product Florajen3^®^ against GBS. Inhibition and complete exclusion of certain GBS strains cocultured with Florajen3^®^ were associated with lower pH values and higher probiotic adhesion affinity to epithelial cells; however, exact pH values were not reported [101]. Patras et al. evaluated nine strains of *Streptococcus salivarius*, the oral commensal, against 13 human GBS isolates. Among the strains tested, K12 exhibited a higher adhesion affinity to both in vitro human and in vivo murine vaginal epithelial cells (VECs) than GBS. Additionally, interleukin-8 levels were lower in human VECs incubated with K12 and GBS compared with VECs incubated with GBS alone [102].

The ability of the studied *Lactobacillus* strains to limit GBS colonization was also observed in vivo in mouse models [103,104,105].

Promising results from in vitro and in vivo studies have led many researchers to focus on evaluating the ability of orally administered probiotic bacteria to reduce vaginal GBS colonization. Scientific studies indicate that women with higher vaginal colonization by lactic acid bacteria are less susceptible to GBS colonization, and oral probiotics may reduce the rates of vaginal and rectal GBS colonization in pregnant women [106,107,108,109]. However, not all proposed strategies are equally effective. It is crucial to develop or identify effective and practical probiotic treatment protocols (optimal doses, timing, and appropriate combinations of probiotic strains) combined with suitable nutrition to prevent or reduce GBS colonization in humans.

Ho et al. obtained promising results when studying the effects of orally administered *Lactobacillus rhamnosus* GR-1 and *Lactobacillus reuteri* RC-14 on GBS colonization in pregnant women with a positive GBS test at 35–37 weeks of gestation [108]. They demonstrated that GBS colonization changed from positive to negative in 42.9% of women taking the probiotics. Similarly, Martin et al. showed that oral probiotics could be an effective method for eradicating GBS from the reproductive tract during pregnancy. Their comprehensive research included isolating *Lactobacillus* strains from the vagina, evaluating their properties, and selecting the best candidate for studies on GBS eradication. They then assessed the efficacy of the selected strain, *L. salivarius*, against GBS in vitro and in vivo using a rat model. Finally, they evaluated the ability of *L. salivarius* CECT 9145 to eradicate GBS from the gastrointestinal and vaginal tracts in pregnant women. In this study, 25 women with a positive GBS test consumed ~9 log_10_ CFU of *L. salivarius* CECT 9145 daily from the 26th to the 38th week of pregnancy. By the study’s end (38th week), 72% and 68% of women in this group were GBS-negative in rectal and vaginal samples, respectively [110].

Farr et al. conducted research to assess the potential of oral probiotics to eliminate GBS colonization during the third trimester of pregnancy [111]. Women with a positive GBS test at 33–37 weeks of gestation were given a dietary supplement containing four live *Lactobacillus* strains (*L. jensenii*, *L. crispatus*, *L. rhamnosus,* and *L. gasseri*) twice daily for 14 days. After treatment, 21/33 (63.6%) women receiving probiotics and 21/27 (77.8%) women in the placebo group remained GBS-positive (*p* = 0.24). Thus, the findings did not confirm the hypothesis that oral probiotics could eliminate GBS during pregnancy. Researchers noted a trend toward lower GBS persistence rates in the probiotic group, but this trend was not statistically significant. Similarly, Sharp et al. found no effect of oral administration of two probiotic strains (*Lactobacillus rhamnosus* GR-1 and *Lactobacillus reuteri* RC-14) on GBS colonization in pregnant women [112]. This study, conducted in Canada with 113 participants (57 receiving probiotics and 56 a placebo), reported no adverse effects in the probiotic group.

Further research is needed to confirm the ability of orally administered probiotics to eradicate vaginal GBS colonization. Future studies should involve double-blind, randomized, controlled trials with larger and more diverse cohorts. Additionally, research aimed at understanding the interactions between GBS and the vaginal microbiome is essential.

### 7.3. Antimicrobial Photodynamic Therapy

In recent years, significant progress has been made in the research on the application of photodynamic inactivation against pathogens. Antimicrobial photodynamic therapy (aPDT) is a promising method used to reduce bacterial colonization. This therapy relies on the synergistic action of light, a photosensitizer, and molecular oxygen present in tissues. The photosensitizer (a chemical substance such as porphyrins, phthalocyanines, or phenothiazines) is applied to the site of GBS colonization, where it binds to the bacteria, including their cell membranes. Irradiation with light of a specific wavelength activates the photosensitizer, causing it to transition to an excited state. The energy is transferred to molecular oxygen, leading to the formation of reactive oxygen species (ROS), such as singlet oxygen or hydroxyl radicals. ROS cause damage to bacterial cell membranes, DNA, and other cellular structures, leading to bacterial death. The effectiveness of aPDT is closely related to the type of photosensitizer and the type of microorganism being targeted.

Preliminary studies indicate that aPDT may reduce the number of viable GBS bacteria on mucosal surfaces, be used in situations where antibiotic therapy is ineffective or contraindicated (e.g., due to allergies), and reduce the risk of GBS transmission from mother to child during childbirth [113,114]. In vitro studies have shown that aPDT is highly effective against GBS, demonstrating a reduction in the number of viable bacteria [115,116]. Pierański et al. observed that aPDT using Bengal rose as a photosensitizer was an effective method for eradicating both planktonic forms and biofilms formed by GBS. They also showed that the therapy did not have mutagenic effects on human cells and did not negatively impact the vaginal microbiota. In in vivo studies (in a mouse model), it was shown that aPDT leads to a significant reduction in GBS survival without significant side effects [117]. However, there are still no large-scale clinical studies to confirm the effectiveness and safety of aPDT in humans in the context of GBS colonization reduction.

The aPDT demonstrates potential in reducing GBS colonization in various locations, including the vagina, without causing resistance, which is a significant advantage over traditional antibiotic therapies. It acts locally, minimizing its impact on the entire microbiome, and when the photosensitizer and light parameters are properly applied, the therapy is safe for host tissues.

Summarizing antimicrobial photodynamic therapy, it is a modern and promising approach to reducing GBS colonization that requires further clinical research to assess its effectiveness and practical applicability.

Advantages and disadvantages of the presented preventive strategies for GBS infections are shown in Table 3.

## 8. Summary

Group B Streptococcus remains one of the leading causes of neonatal morbidity and mortality worldwide. We acknowledge IAP as clinically effective and evidence-based, particularly in reducing the early-onset neonatal GBS infections. However, IAP is incomplete as a long-term solution because it fails to prevent late-onset disease, may accelerate antibiotic resistance, and negatively affect the neonatal microbiota, potentially leading to undesirable long-term health consequences. Maternal colonization remains the primary risk factor for neonatal GBS infection, highlighting the need for effective and sustainable strategies to prevent mother-to-child transmission.

GBS vaccine development as a public health priority should be emphasized. A successful vaccine could eliminate the need for IAP, reduce the risk of both early- and late-onset infections, and provide protection in regions where universal screening is difficult to implement. Clinical trials have shown promising results, but regulatory approval is still pending.

Recent advances in our understanding of the vaginal microbiota and host–pathogen interactions have opened new possibilities for GBS prevention. Probiotic interventions, particularly those involving selected *Lactobacillus* strains—dominant species in a healthy vaginal microbiota—show promise in modulating the vaginal ecosystem and reducing GBS colonization. Special attention has been given to *L. crispatus*, *L. gasseri*, and *L. rhamnosus*, which are considered key to maintaining mucosal balance. While we recognize the benefits, we emphasize that current clinical evidence is inconsistent. Therefore, large-scale, randomized controlled trials should be conducted before probiotics can be considered reliable or standardized.

In addition to vaccines and probiotics, other experimental approaches to GBS prevention include antimicrobial photodynamic therapy, an innovative, non-antibiotic alternative that allows for localized GBS eradication without inducing resistance.

## 9. Future Directions

Future research should focus on developing preventive strategies that are effective and safe for both mothers and neonates while addressing global disparities in access to care.

Completion of Phase III trials for multivalent conjugate and protein-based vaccines is essential to determine their efficacy, durability of protection, and effectiveness in diverse geographic settings. Continued genomic surveillance is necessary to monitor serotype shifts and to guide vaccine updates.

Although certain *Lactobacillus* strains show potential in reducing GBS colonization, basic research results are inconsistent. Future studies should point to optimal strains, doses, and treatment protocols, supported by multi-omics analyses to better understand microbiome dynamics and predict treatment response.

Antimicrobial photodynamic therapy represents a promising alternative treatment for local GBS eradication without inducing bacterial resistance. Further preclinical and clinical trials are needed to assess its safety, justification for application, and integration into prenatal care.

Integrating multiple preventive approaches and ensuring their accessibility will be essential for reducing the global burden of neonatal GBS diseases.

## Figures and Tables

**Table 1:** Distribution of the most prevalent GBS serotypes.

Most Prevalent Serotype *	Region	References
III, Ia, Ib, V, II, IV and other serotypes **	Europe	[16,17,18,19]
Ia and Ib, V, III, IV	North America	[19]
Ia and Ib, II, III	Central America	[19]
Ia and Ib, III, II, V and other serotypes	South America	[19]
Ia and Ib, III, V, II, IV and other serotypes, IV and Ia	Western Asia (United Arab Emirates)	[19]
Ia and Ib, III, II, V, other serotypes and IV Ia and Ib, III	Southern Asia (China)	[19,20]
V, Ia and Ib, other serotypes, III and IV	Southeastern Asia	[19]
Ia and Ib, III, V, other serotypes and II	Estern Asia	[19]
Ia and Ib, V, III and II	Middle Africa	[19]
V, Ia and Ib, III and II V	Western Africa (Egypt)	[19,21]
III, V, Ia and Ib, II and IV	Eastern Africa	[19]
Ia and Ib, III, V, II and IV	Southern Africa	[19]
Ia and Ib, III, V, II, IV and other serotypes	Australia and New Zealand	[19]

* The order in which serotypes are listed indicates decreasing frequency of occurrence (based on the cited references). ** Other serotypes—VI, VII, VIII, IX (Serotype VIII is rarely identified outside Japan).

**Table 2:** The main GBS virulence factors, with their specific targets and function.

Virulence Factor	Specific Target	Function/Characteristics	References
Fibrinogen binding protein A (FbsA)	Fibrinogen	Adhesion to endothelial and epithelial cells; Anti-phagocytic activity; Aggregation of platelets	[29,43]
Fibrinogen binding protein B (FbsB)	Fibrinogen	Invasion	[29,43]
Fibrinogen binding protein C (FbsC)	Fibrinogen	Attachment to and invasion of epithelial and endothelial barriers; Biofilm formation	[29,43]
Laminin binding protein (Lmb)	Laminin	Colonize and invade host epithelium; Neurotropism	[28,31]
Plasminogen binding surface protein (PbsP)	Plasminogen	Extracellular proteolytic activity; Transmigration across brain endothelial cells	[44]
Serine-rich repeat protein 1 and 2 (Srr1 and Srr2)	Fibrinogen	Adherence to vaginal and cervical epithelial cells	[2,30]
GBS immunogenic bacterial adhesin (BibA)	C4-binding protein	Disruption of complement component C4 binding; Antiphagocytic activity	[32]
GBS surface adhesin (BsaB)	Fibronectin, laminin	Attachment to epithelial cells; Biofilm formation	[29]
C5a peptidase (ScpB)	C5a fibronectin	Cleavage of C5a and inhibition of neutrophil recruitment to the infection site; Adherence/invasion to epithelial cells	[45]
Hypervirulent GBS adhesin (HvgA)	Unknown	Attachment to endothelial and epithelial cells; Meningeal tropism; Specific for St-17 clone	[33]
Pili	Collagen I	Evasion of innate immunity; Macrophage intracellular survival; Penetration of blood–brain barrier; Biofilm formation	[36,37,38]
Alpha C protein (ACP)	Glycosaminoglycans	Invasion of cervical epithelial cells	[46]
GBS hyaluronidase (HylB)	Hyaluronic acid	Inhibition of ROS production; Resistance of neutrophils	[2,9]
Hemolytic pigment	Neutrophils, mast cells, and macrophages	Resistance to mast cells, macrophages, and neutrophils; Penetration of the placenta; Invasion of the amniotic cavity; Kills platelets	[47]
Capsular polysaccharides (CPS)	Siglecs	Defense from host deposition; Inhibition of the activation of neutrophils and macrophages; Resistance to platelets	[2,14]

**Table 3:** Comparison of preventive strategies against GBS.

Type of Prevention	Advantages	Disadvantages
Vaccination	- Passive protection of the newborn by maternal antibodies (IgG) - Possibility of preventing the early and late GBS infections - Reducing the use of antibiotic prophylaxis - No adverse effect on the newborn’s microbiota - Preventing GBS infections in the low- and middle-income countries	- No registered and/or available vaccines against GBS infections - Lack of unified rules for vaccine licensing-unknown interference with other vaccines administered during pregnancy, to be estimated - Serotype limitations (risk of incomplete coverage)
Probiotics	- Potential reduction in GBS colonization in the vagina - Do not generate antibiotic resistance - Safe for mother and child and well tolerated	- No treatment protocols (lack of standardization of strains, doses and duration of administration) - Inconclusive clinical efficacy
aPDT	- Reduction in both planktonic and biofilm forms on mucosal surfaces - Local implementation and action - No negative impact on the beneficial microbiota - No significant side effects - No induction of bacterial resistance	- Lack of large-scale clinical trials - Limited number of assessments in pregnant women - It requires specialized equipment and appropriately selected photosensitizers

## Data Availability

No new data were created or analyzed in this study. Data sharing is not applicable to this article.

## References

[1] Raabe V.N., Shane A.L. (2019). Group B *Streptococcus* (*Streptococcus agalactiae*). Microbiol. Spectr..

[2] Armistead B., Oler E., Adams Waldorf K., Rajagopal L. (2019). The Double Life of Group B Streptococcus: Asymptomatic Colonizer and Potent Pathogen. J. Mol. Biol..

[3] Verani J.R., McGee L., Schrag S.J., Division of Bacterial Diseases, National Center for Immunization and Respiratory Diseases, Centers for Disease Control and Prevention (CDC) (2010). Prevention of perinatal group B streptococcal disease—Revised guidelines from CDC, 2010. MMWR Recomm. Rep..

[4] Naat G., Encourage P. (2020). Prevention of Group B Streptococcal Early-Onset Disease in Newborns: ACOG Committee Opinion, Number 797. Obstet. Gynecol..

[5] Rosini R., Margarit I. (2015). Biofilm formation by *Streptococcus agalactiae*: Influence of environmental conditions and implicated virulence factors. Front. Cell. Infect. Microbiol..

[6] Puopolo K.M., Lynfield R., Cummings J.J., Hand I., Adams-Chapman I., Poindexter B., Stewart D.L., Aucott S.W., Committee on Fetus and Newborn, Committee on Infectious Diseases (2019). Management of Infants at Risk for Group B Streptococcal Disease. Pediatrics.

[7] Le Doare K., Heath P.T. (2013). An overview of global GBS epidemiology. Vaccine.

[8] Miselli F., Frabboni I., Di Martino M., Zinani I., Buttera M., Insalaco A., Stefanelli F., Lugli L., Berardi A. (2022). Transmission of Group B Streptococcus in late-onset neonatal disease: A narrative review of current evidence. Ther. Adv. Infect. Dis..

[9] Burcham L.R., Spencer B.L., Keeler L.R., Runft D.L., Patras K.A., Neely M.N., Doran K.S. (2019). Determinants of Group B streptococcal virulence potential amongst vaginal clinical isolates from pregnant women. PLoS ONE.

[10] Bartlett A.W., Smith B., George C.R.R., McMullan B., Kesson A., Lahra M.M., Palasanthiran P. (2017). Epidemiology of Late and Very Late Onset Group B Streptococcal Disease: Fifteen-Year Experience From Two Australian Tertiary Pediatric Facilities. Pediatr. Infect. Dis. J..

[11] Guilbert J., Levy C., Cohen R., Delacourt C., Renolleau S., Flamant C., Bacterial Meningitis Group (2010). Late and ultra late onset *Streptococcus B* meningitis: Clinical and bacteriological data over 6 years in France. Acta Paediatr..

[12] Burianová I., Paulová M., Čermák P., Janota J. (2013). Group B *Streptococcus* Colonization of Breast Milk of Group B *Streptococcus* Positive Mothers. J. Hum. Lact..

[13] Freudenhammer M., Karampatsas K., Le Doare K., Lander F., Armann J., Acero Moreno D., Boyle M., Buxmann H., Campbell R., Chalker V. (2021). Invasive Group B Streptococcus Disease with Recurrence and in Multiples: Towards a Better Understanding of GBS Late-Onset Sepsis. Front. Immunol..

[14] Shabayek S., Spellerberg B. (2018). Group B Streptococcal Colonization, Molecular Characteristics, and Epidemiology. Front. Microbiol..

[15] Slotved H.-C., Kong F., Lambertsen L., Sauer S., Gilbert G.L. (2007). Serotype IX, a Proposed New *Streptococcus agalactiae* Serotype. J. Clin. Microbiol..

[16] Ippolito D.L., James W.A., Tinnemore D., Huang R.R., Dehart M.J., Williams J., Wingerd M.A., Demons S.T. (2010). Group B streptococcusserotype prevalence in reproductive-age women at a tertiary care military medical center relative to global serotype distribution. BMC Infect. Dis..

[17] Lamagni T.L., Keshishian C., Efstratiou A., Guy R., Henderson K.L., Broughton K., Sheridan E. (2013). Emerging Trends in the Epidemiology of Invasive Group B Streptococcal Disease in England and Wales, 1991–2010. Clin. Infect. Dis..

[18] Florindo C., Damião V., Silvestre I., Farinha C., Rodrigues F., Nogueira F., Martins-Pereira F., Castro R., Borrego M.J., Santos-Sanches I. (2014). Epidemiological surveillance of colonising group B Streptococcus epidemiology in the Lisbon and Tagus Valley regions, Portugal (2005 to 2012): Emergence of a new epidemic type IV/clonal complex 17 clone. Eurosurveillance.

[19] Russell N.J., Seale A.C., O’Driscoll M., O’Sullivan C., Bianchi-Jassir F., Gonzalez-Guarin J., Lawn J.E., Baker C.J., Bartlett L., Cutland C. (2017). Maternal Colonization with Group B Streptococcus and Serotype Distribution Worldwide: Systematic Review and Meta-analyses. Clin. Infect. Dis..

[20] Chen Y., Liu L., Liu J., Ji T., Gao Y., Yang D., Zhao M., Zhai Y., Cao Z. (2024). Serotype distribution, antimicrobial resistance, and molecular characterization of group B *Streptococcus* isolates from Chinese pregnant woman. J. Matern. Fetal Neonatal Med..

[21] Shabayek S., Abdalla S., Abouzeid A.M. (2014). Serotype and surface protein gene distribution of colonizing group B streptococcus in women in Egypt. Epidemiol. Infect..

[22] Madrid L., Seale A.C., Kohli-Lynch M., Edmond K.M., Lawn J.E., Heath P.T., Madhi S.A., Baker C.J., Bartlett L., Cutland C. (2017). Infant Group B Streptococcal Disease Incidence and Serotypes Worldwide: Systematic Review and Meta-analyses. Clin. Infect. Dis..

[23] Weisner A.M., Johnson A.P., Lamagni T.L., Arnold E., Warner M., Heath P.T., Efstratiou A. (2004). Characterization of Group B Streptococci Recovered from Infants with Invasive Disease in England and Wales. Clin. Infect. Dis..

[24] Salloum M., Van Der Mee-Marquet N., Valentin-Domelier A.-S., Quentin R. (2011). Diversity of Prophage DNA Regions of *Streptococcus agalactiae* Clonal Lineages from Adults and Neonates with Invasive Infectious Disease. PLoS ONE.

[25] Perme T., Golparian D., Bombek Ihan M., Rojnik A., Lučovnik M., Kornhauser Cerar L., Fister P., Lozar Krivec J., Grosek Š., Ihan A. (2020). Genomic and phenotypic characterisation of invasive neonatal and colonising group B Streptococcus isolates from Slovenia, 2001–2018. BMC Infect. Dis..

[26] Creti R., Imperi M., Berardi A., Lindh E., Alfarone G., Pataracchia M., Recchia S. (2021). The Italian Network on Neonatal and Infant GBS Infections Invasive Group B Streptococcal Disease in Neonates and Infants, Italy, Years 2015–2019. Microorganisms.

[27] Brzychczy-Włoch M., Gosiewski T., Pawlik D., Szumała-Kakol A., Samead A., Heczko P.B. (2012). Occurrence of the hypervirulent ST-17 clone of *Streptococcus agalactiae* in pregnant women and newborns. Przegl. Epidemiol..

[28] Kamińska D., Ratajczak M., Nowak-Malczewska D.M., Karolak J.A., Kwaśniewski M., Szumala-Kakol A., Dlugaszewska J., Gajecka M. (2024). Macrolide and lincosamide resistance of *Streptococcus agalactiae* in pregnant women in Poland. Sci. Rep..

[29] Jiang S., Wessels M.R. (2014). BsaB, a Novel Adherence Factor of Group B Streptococcus. Infect. Immun..

[30] Six A., Bellais S., Bouaboud A., Fouet A., Gabriel C., Tazi A., Dramsi S., Trieu-Cuot P., Poyart C. (2015). Srr2, a multifaceted adhesin expressed by ST-17 hypervirulent Group B *Streptococcus* involved in binding to both fibrinogen and plasminogen. Mol. Microbiol..

[31] Al Safadi R., Amor S., Hery-Arnaud G., Spellerberg B., Lanotte P., Mereghetti L., Gannier F., Quentin R., Rosenau A. (2010). Enhanced Expression of lmb Gene Encoding Laminin-Binding Protein in *Streptococcus agalactiae* Strains Harboring IS1548 in scpB-lmb Intergenic Region. PLoS ONE.

[32] Santi I., Maione D., Galeotti C.L., Grandi G., Telford J.L., Soriani M. (2009). BibA Induces Opsonizing Antibodies Conferring In Vivo Protection against Group B *Streptococcus*. J. Infect. Dis..

[33] Tazi A., Disson O., Bellais S., Bouaboud A., Dmytruk N., Dramsi S., Mistou M.-Y., Khun H., Mechler C., Tardieux I. (2010). The surface protein HvgA mediates group B streptococcus hypervirulence and meningeal tropism in neonates. J. Exp. Med..

[34] Plainvert C., Hays C., Touak G., Joubrel-Guyot C., Dmytruk N., Frigo A., Poyart C., Tazi A. (2020). Multidrug-Resistant Hypervirulent Group B *Streptococcus* in Neonatal Invasive Infections, France, 2007–2019. Emerg. Infect. Dis..

[35] Martins E.R., Pedroso-Roussado C., Melo-Cristino J., Ramirez M., The Portuguese Group for the Study of Streptococcal Infections (2017). *Streptococcus agalactiae* Causing Neonatal Infections in Portugal (2005–2015): Diversification and Emergence of a CC17/PI-2b Multidrug Resistant Sublineage. Front. Microbiol..

[36] Nobbs A.H., Rosini R., Rinaudo C.D., Maione D., Grandi G., Telford J.L. (2008). Sortase A Utilizes an Ancillary Protein Anchor for Efficient Cell Wall Anchoring of Pili in *Streptococcus agalactiae*. Infect. Immun..

[37] Margarit I., Rinaudo C.D., Galeotti C.L., Maione D., Ghezzo C., Buttazzoni E., Rosini R., Runci Y., Mora M., Buccato S. (2009). Preventing Bacterial Infections with Pilus-Based Vaccines: The Group B Streptococcus Paradigm. J. Infect. Dis..

[38] Springman A.C., Lacher D.W., Waymire E.A., Wengert S.L., Singh P., Zadoks R.N., Davies H.D., Manning S.D. (2014). Pilus distribution among lineages of group b streptococcus: An evolutionary and clinical perspective. BMC Microbiol..

[39] Wang J., Li W., Li N., Wang B. (2023). Immunization with Multiple Virulence Factors Provides Maternal and Neonatal Protection against Group B Streptococcus Serotypes. Vaccines.

[40] Rajagopal L. (2009). Understanding the Regulation of Group B *Streptococcal* Virulence Factors. Future Microbiol..

[41] Liu Y., Liu J. (2022). Group B *Streptococcus*: Virulence Factors and Pathogenic Mechanism. Microorganisms.

[42] Megli C.J., Carlin S.M., Giacobe E.J., Hillebrand G.H., Hooven T.A. (2025). Virulence and pathogenicity of group B *Streptococcus*: Virulence factors and their roles in perinatal infection. Virulence.

[43] Buscetta M., Papasergi S., Firon A., Pietrocola G., Biondo C., Mancuso G., Midiri A., Romeo L., Teti G., Speziale P. (2014). FbsC, a Novel Fibrinogen-binding Protein, Promotes *Streptococcus agalactiae*-Host Cell Interactions. J. Biol. Chem..

[44] Buscetta M., Firon A., Pietrocola G., Biondo C., Mancuso G., Midiri A., Romeo L., Galbo R., Venza M., Venza I. (2016). PbsP, a cell wall-anchored protein that binds plasminogen to promote hematogenous dissemination of group B *Streptococcus*. Mol. Microbiol..

[45] Hull J.R., Tamura G.S., Castner D.G. (2008). Interactions of the streptococcal C5a peptidase with human fibronectin. Acta Biomater..

[46] Baron M.J., Filman D.J., Prophete G.A., Hogle J.M., Madoff L.C. (2007). Identification of a Glycosaminoglycan Binding Region of the Alpha C Protein That Mediates Entry of Group B *Streptococci* into Host Cells. J. Biol. Chem..

[47] Whidbey C., Harrell M.I., Burnside K., Ngo L., Becraft A.K., Iyer L.M., Aravind L., Hitti J., Adams Waldorf K.M., Rajagopal L. (2013). A hemolytic pigment of Group B *Streptococcus* allows bacterial penetration of human placenta. J. Exp. Med..

[48] Abranches J., Zeng L., Kajfasz J.K., Palmer S.R., Chakraborty B., Wen Z.T., Richards V.P., Brady L.J., Lemos J.A. (2018). Biology of Oral Streptococci. Microbiol. Spectr..

[49] Rinaudo C.D., Rosini R., Galeotti C.L., Berti F., Necchi F., Reguzzi V., Ghezzo C., Telford J.L., Grandi G., Maione D. (2010). Specific Involvement of Pilus Type 2a in Biofilm Formation in Group B *Streptococcus*. PLoS ONE.

[50] Konto-Ghiorghi Y., Mairey E., Mallet A., Duménil G., Caliot E., Trieu-Cuot P., Dramsi S. (2009). Dual Role for Pilus in Adherence to Epithelial Cells and Biofilm Formation in *Streptococcus agalactiae*. PLoS Pathog..

[51] Nie S., Lu X., Hu Y.-W., Zheng L., Wang Q. (2018). Influence of environmental and genotypic factors on biofilm formation by clinical isolates of group B streptococci. Microb. Pathog..

[52] D’Urzo N., Martinelli M., Pezzicoli A., De Cesare V., Pinto V., Margarit I., Telford J.L., Maione D. (2014). Acidic pH Strongly Enhances In Vitro Biofilm Formation by a Subset of Hypervirulent ST-17 *Streptococcus agalactiae* Strains. Appl. Environ. Microbiol..

[53] Sabroske E.M., Iglesias M.A.S., Rench M., Moore T., Harvey H., Edwards M., Baker C.J., Flores A.R. (2023). Evolving antibiotic resistance in Group B Streptococci causing invasive infant disease: 1970–2021. Pediatr. Res..

[54] Emaneini M., Mirsalehian A., Beigvierdi R., Fooladi A.A.I., Asadi F., Jabalameli F., Taherikalani M. (2014). High Incidence of Macrolide and Tetracycline Resistance Among *Streptococcus agalactiae* Strains Isolated from Clinical Samples in Tehran, Iran. Maedica.

[55] Berbel D., González-Díaz A., De Egea G.L., Càmara J., Ardanuy C. (2022). An Overview of Macrolide Resistance in Streptococci: Prevalence, Mobile Elements and Dynamics. Microorganisms.

[56] Alzayer M., Alkhulaifi M.M., Alyami A., Aldosary M., Alageel A., Garaween G., Shibl A., Al-Hamad A.M., Doumith M. (2023). Molecular typing and antimicrobial resistance of group B Streptococcus clinical isolates in Saudi Arabia. J. Glob. Antimicrob. Resist..

[57] Genovese C., D’Angeli F., Di Salvatore V., Tempera G., Nicolosi D. (2020). *Streptococcus agalactiae* in pregnant women: Serotype and antimicrobial susceptibility patterns over five years in Eastern Sicily (Italy). Eur. J. Clin. Microbiol. Infect. Dis..

[58] Bolukaoto J.Y., Monyama C.M., Chukwu M.O., Lekala S.M., Nchabeleng M., Maloba M.R.B., Mavenyengwa R.T., Lebelo S.L., Monokoane S.T., Tshepuwane C. (2015). Antibiotic resistance of *Streptococcus agalactiae* isolated from pregnant women in Garankuwa, South Africa. BMC Res. Notes.

[59] Cox L.M., Yamanishi S., Sohn J., Alekseyenko A.V., Leung J.M., Cho I., Kim S.G., Li H., Gao Z., Mahana D. (2014). Altering the Intestinal Microbiota during a Critical Developmental Window Has Lasting Metabolic Consequences. Cell.

[60] Gajecka M., Gutaj P., Jaskiewicz K., Rydzanicz M., Szczapa T., Kaminska D., Kosewski G., Przyslawski J., Ploski R., Wender-Ozegowska E. (2024). Effects of maternal type 1 diabetes and confounding factors on neonatal microbiomes. Diabetologia.

[61] Dierikx T.H., Visser D.H., Benninga M.A., Van Kaam A.H.L.C., De Boer N.K.H., De Vries R., Van Limbergen J., De Meij T.G.J. (2020). The influence of prenatal and intrapartum antibiotics on intestinal microbiota colonisation in infants: A systematic review. J. Infect..

[62] Prescott S., Dreisbach C., Baumgartel K., Koerner R., Gyamfi A., Canellas M., St. Fleur A., Henderson W.A., Trinchieri G. (2021). Impact of Intrapartum Antibiotic Prophylaxis on Offspring Microbiota. Front. Pediatr..

[63] Diamond L., Wine R., Morris S.K. (2022). Impact of intrapartum antibiotics on the infant gastrointestinal microbiome: A narrative review. Arch. Dis. Child..

[64] Corvaglia L., Tonti G., Martini S., Aceti A., Mazzola G., Aloisio I., Di Gioia D., Faldella G. (2016). Influence of Intrapartum Antibiotic Prophylaxis for Group B *Streptococcus* on Gut Microbiota in the First Month of Life. J. Pediatr. Gastroenterol. Nutr..

[65] Carreras-Abad C., Ramkhelawon L., Heath P.T., Le Doare K. (2020). A Vaccine Against Group B Streptococcus: Recent Advances. Infect. Drug Resist..

[66] Pena J.M.S., Lannes-Costa P.S., Nagao P.E. (2024). Vaccines for *Streptococcus agalactiae*: Current status and future perspectives. Front. Immunol..

[67] Procter S.R., Gonçalves B.P., Paul P., Chandna J., Seedat F., Koukounari A., Hutubessy R., Trotter C., Lawn J.E., Jit M. (2023). Maternal immunisation against Group B Streptococcus: A global analysis of health impact and cost-effectiveness. PLoS Med..

[68] Paul P., Gonçalves B.P., Le Doare K., Lawn J.E. (2023). 20 million pregnant women with group B streptococcus carriage: Consequences, challenges, and opportunities for prevention. Curr. Opin. Pediatr..

[69] Madhi S.A., Anderson A.S., Absalon J., Radley D., Simon R., Jongihlati B., Strehlau R., Van Niekerk A.M., Izu A., Naidoo N. (2023). Potential for Maternally Administered Vaccine for Infant Group B Streptococcus. N. Engl. J. Med..

[70] Furfaro L.L., Chang B.J., Payne M.S. (2018). Perinatal *Streptococcus agalactiae* Epidemiology and Surveillance Targets. Clin. Microbiol. Rev..

[71] Kim S.-Y., Nguyen C., Russell L.B., Tomczyk S., Abdul-Hakeem F., Schrag S.J., Verani J.R., Sinha A. (2017). Cost-effectiveness of a potential group B streptococcal vaccine for pregnant women in the United States. Vaccine.

[72] Baker C.J., Kasper D.L. (1976). Correlation of Maternal Antibody Deficiency with Susceptibility to Neonatal Group B Streptococcal Infection. N. Engl. J. Med..

[73] Sebghati M., Khalil A. (2021). Uptake of vaccination in pregnancy. Best Pract. Res. Clin. Obstet. Gynaecol..

[74] Gupalova T., Leontieva G., Kramskaya T., Grabovskaya K., Bormotova E., Korjevski D., Suvorov A. (2018). Development of experimental GBS vaccine for mucosal immunization. PLoS ONE.

[75] Bedeley E., Gori A., Yeboah-Manu D., Diallo K. (2021). Control of Streptococcal Infections: Is a Common Vaccine Target Achievable Against *Streptococcus agalactiae* and Streptococcus pneumoniae. Front. Microbiol..

[76] Smith W.B., Seger W., Chawana R., Skogeby Z., Silmon De Monerri N.C., Feng Y., Gaylord M., Jongihlati B., Beeslaar J., Skinner J.M. (2025). A Phase 2b Trial Evaluating the Safety, Tolerability, and Immunogenicity of a 6-Valent Group B *Streptococcus* Vaccine Administered Concomitantly with Tetanus, Diphtheria, and Acellular Pertussis Vaccine in Healthy Nonpregnant Female Individuals. J. Infect. Dis..

[77] Pell M.E., McCutcheon C.R., Gaddy J.A., Aronoff D.M., Petroff M.G., Manning S.D. (2025). Impact of antibiotics on membrane vesicle production in Group B *Streptococcus*. Microbiol. Spectr..

[78] Pfizer (2024). A Phase 1/2, Randomized, Placebo-Controlled, Observer-Blinded Trial to Evaluate the Safety, Tolerability, and Immunogenicity of a Multivalent Group B Streptococcus Vaccine in Healthy Nonpregnant Women and Pregnant Women 18 to 40 Years of Age and Their Infants. Clinicaltrials.Gov; Report No.: NCT03765073. NCT03765073.

[79] Lin S.-M., Jang A.-Y., Zhi Y., Gao S., Lim S., Lim J.H., Song J.Y., Sullam P.M., Rhee J.H., Seo H.S. (2018). Vaccination with a Latch Peptide Provides Serotype-Independent Protection Against Group B Streptococcus Infection in Mice. J. Infect. Dis..

[80] Trotter C.L., Alderson M., Dangor Z., Ip M., Le Doare K., Nakabembe E., Procter S.R., Sekikubo M., Lambach P. (2023). Vaccine value profile for Group B streptococcus. Vaccine.

[81] Sharp M.E., Sproch J., Haldeman S., Tettelin H., Ratner A.J. (2025). Expansion of the Group B *Streptococcus* serotype repertoire via gene acquisition from other streptococcal species. Microbiol. Spectr..

[82] Projekt JUNO. https://www.gbsgen.net/.

[83] Zhou X., Hansmann M.A., Davis C.C., Suzuki H., Brown C.J., Schütte U., Pierson J.D., Forney L.J. (2010). The vaginal bacterial communities of Japanese women resemble those of women in other racial groups. FEMS Immunol. Med. Microbiol..

[84] Ravel J., Gajer P., Abdo Z., Schneider G.M., Koenig S.S.K., McCulle S.L., Karlebach S., Gorle R., Russell J., Tacket C.O. (2011). Vaginal microbiome of reproductive-age women. Proc. Natl. Acad. Sci. USA.

[85] Benschop C.C.G., Quaak F.C.A., Boon M.E., Sijen T., Kuiper I. (2012). Vaginal microbial flora analysis by next generation sequencing and microarrays; can microbes indicate vaginal origin in a forensic context?. Int. J. Leg. Med..

[86] Kamińska D., Gajecka M. (2017). Is the role of human female reproductive tract microbiota underestimated?. Benef. Microbes.

[87] Tachedjian G., Aldunate M., Bradshaw C.S., Cone R.A. (2017). The role of lactic acid production by probiotic Lactobacillus species in vaginal health. Res. Microbiol..

[88] Petrova M.I., Lievens E., Verhoeven T.L.A., Macklaim J.M., Gloor G., Schols D., Vanderleyden J., Reid G., Lebeer S. (2016). The lectin-like protein 1 in *Lactobacillus rhamnosus* GR-1 mediates tissue-specific adherence to vaginal epithelium and inhibits urogenital pathogens. Sci. Rep..

[89] Allonsius C.N., Van Den Broek M.F.L., De Boeck I., Kiekens S., Oerlemans E.F.M., Kiekens F., Foubert K., Vandenheuvel D., Cos P., Delputte P. (2017). Interplay between *Lactobacillus rhamnosus* GG and *Candida* and the involvement of exopolysaccharides. Microb. Biotechnol..

[90] Liu P., Lu Y., Li R., Chen X. (2023). Use of probiotic lactobacilli in the treatment of vaginal infections: In Vitro and in vivo investigations. Front. Cell. Infect. Microbiol..

[91] Avitabile E., Menotti L., Croatti V., Giordani B., Parolin C., Vitali B. (2024). Protective Mechanisms of Vaginal Lactobacilli against Sexually Transmitted Viral Infections. Int. J. Mol. Sci..

[92] Wasfi R., Abd El-Rahman O.A., Zafer M.M., Ashour H.M. (2018). Probiotic *Lactobacillus* sp. inhibit growth, biofilm formation and gene expression of caries-inducing *Streptococcus mutans*. J. Cell. Mol. Med..

[93] Shiroda M., Aronoff D.M., Gaddy J.A., Manning S.D. (2020). The impact of Lactobacillus on group B streptococcal interactions with cells of the extraplacental membranes. Microb. Pathog..

[94] Giordani B., Naldi M., Croatti V., Parolin C., Erdoğan Ü., Bartolini M., Vitali B. (2023). Exopolysaccharides from vaginal lactobacilli modulate microbial biofilms. Microb. Cell Factories.

[95] Kalia N., Singh J., Kaur M. (2020). Microbiota in vaginal health and pathogenesis of recurrent vulvovaginal infections: A critical review. Ann. Clin. Microbiol. Antimicrob..

[96] Marziali G., Foschi C., Parolin C., Vitali B., Marangoni A. (2019). In-vitro effect of vaginal lactobacilli against group B Streptococcus. Microb. Pathog..

[97] Zarate G., Nader-Macias M.E. (2006). Influence of probiotic vaginal lactobacilli on in vitro adhesion of urogenital pathogens to vaginal epithelial cells. Lett. Appl. Microbiol..

[98] Ortiz L., Ruiz F., Pascual L., Barberis L. (2014). Effect of Two Probiotic Strains of Lactobacillus on In Vitro Adherence of Listeria monocytogenes, *Streptococcus agalactiae*, and Staphylococcus aureus to Vaginal Epithelial Cells. Curr. Microbiol..

[99] Leccese Terraf M.C., Mendoza L.M., Juárez Tomás M.S., Silva C., Nader-Macías M.E.F. (2014). Phenotypic surface properties (aggregation, adhesion and biofilm formation) and presence of related genes in beneficial vaginal *lactobacilli*. J. Appl. Microbiol..

[100] Ventolini G. (2015). Vaginal Lactobacillus: Biofilm formation in vivo—Clinical implications. Int. J. Womens Health.

[101] Ephraim E., Schultz R., Duster-Matz M., Warrack S., Spiegel C., Safdar N. (2012). In-Vitro evaluation of the antagonistic effects of the probiotics *Lactobacillus rhamnosus* HN001 and Florajen 3 against group B Streptococci. Int. J. Probiotics Prebiotics.

[102] Patras K.A., Wescombe P.A., Rösler B., Hale J.D., Tagg J.R., Doran K.S. (2015). Streptococcus salivarius K12 Limits Group B Streptococcus Vaginal Colonization. Infect. Immun..

[103] De Gregorio P.R., Juárez Tomás M.S., Leccese Terraf M.C., Nader-Macías M.E.F. (2015). Preventive effect of *Lactobacillus reuteri* CRL1324 on Group B *Streptococcus* vaginal colonization in an experimental mouse model. J. Appl. Microbiol..

[104] De Gregorio P.R., Juárez Tomás M.S., Nader-Macías M.E.F. (2016). Immunomodulation of *Lactobacillus reuteri* CRL1324 on Group B *Streptococcus* Vaginal Colonization in a Murine Experimental Model. Am. J. Reprod. Immunol..

[105] Patras K.A., Doran K.S. (2016). A Murine Model of Group B Streptococcus Vaginal Colonization. J. Vis. Exp..

[106] Rosen G.H., Randis T.M., Desai P.V., Sapra K.J., Ma B., Gajer P., Humphrys M.S., Ravel J., Gelber S.E., Ratner A.J. (2017). Group B Streptococcus and the Vaginal Microbiota. J. Infect. Dis..

[107] Whitney C., Daly S., Limpongsanurak S., Festin M., Thinn K., Chipato T., Lumbiganon P., Sauvarin J., Andrews W., Tolosa J. (2004). The International Infections in Pregnancy Study: Group B streptococcal colonization in pregnant women. J. Matern. Fetal Neonatal Med..

[108] Ho M., Chang Y.-Y., Chang W.-C., Lin H.-C., Wang M.-H., Lin W.-C., Chiu T.-H. (2016). Oral *Lactobacillus rhamnosus* GR-1 and *Lactobacillus reuteri* RC-14 to reduce Group B Streptococcus colonization in pregnant women: A randomized controlled trial. Taiwan. J. Obstet. Gynecol..

[109] Hanson L., VandeVusse L., Forgie M., Malloy E., Singh M., Scherer M., Kleber D., Dixon J., Hryckowian A.J., Safdar N. (2023). A randomized controlled trial of an oral probiotic to reduce antepartum group B Streptococcus colonization and gastrointestinal symptoms. Am. J. Obstet. Gynecol. MFM.

[110] Martín V., Cárdenas N., Ocaña S., Marín M., Arroyo R., Beltrán D., Badiola C., Fernández L., Rodríguez J.M. (2019). Rectal and Vaginal Eradication of *Streptococcus agalactiae* (GBS) in Pregnant Women by Using *Lactobacillus salivarius* CECT 9145, A Target-specific Probiotic Strain. Nutrients.

[111] Farr A., Sustr V., Kiss H., Rosicky I., Graf A., Makristathis A., Foessleitner P., Petricevic L. (2020). Oral probiotics to reduce vaginal group B streptococcal colonization in late pregnancy. Sci. Rep..

[112] Sharpe M., Shah V., Freire-Lizama T., Cates E.C., McGrath K., David I., Cowan S., Letkeman J., Stewart-Wilson E. (2021). Effectiveness of oral intake of *Lactobacillus rhamnosus GR-1* and *Lactobacillus reuteri RC-14* on Group B *Streptococcus* colonization during pregnancy: A midwifery-led double-blind randomized controlled pilot trial. J. Matern. Fetal Neonatal Med..

[113] Yi M., Wang H., Wang M., Cao J., Gao F., Ke X., Liu Z., Liu Y., Lu M. (2021). Efficient Inhibition of *Streptococcus agalactiae* by AIEgen-Based Fluorescent Nanomaterials. Front. Chem..

[114] Sellera F.P., Sabino C.P., Ribeiro M.S., Gargano R.G., Benites N.R., Melville P.A., Pogliani F.C. (2016). In Vitro photoinactivation of bovine mastitis related pathogens. Photodiagnosis Photodyn. Ther..

[115] Pieranski M., Sitkiewicz I., Grinholc M. (2020). Increased photoinactivation stress tolerance of *Streptococcus agalactiae* upon consecutive sublethal phototreatments. Free Radic. Biol. Med..

[116] Pieranski M.K., Rychlowski M., Grinholc M. (2021). Optimization of *Streptococcus agalactiae* Biofilm Culture in a Continuous Flow System for Photoinactivation Studies. Pathogens.

[117] Pierański M.K., Kosiński J.G., Szymczak K., Sadowski P., Grinholc M. (2023). Antimicrobial Photodynamic Inactivation: An Alternative for Group B Streptococcus Vaginal Colonization in a Murine Experimental Model. Antioxidants.

